# A weighted sentiment dataset for Indonesian telemedicine text classification

**DOI:** 10.1016/j.dib.2026.113172

**Published:** 2026-08-26

**Authors:** Fakhri Wahid Athallah, Ihya Choerul Ulumudin, Rhio Sutoyo, Esther Widhi Andangsari

**Affiliations:** a Computer Science Department, School of Computer Science, Bina Nusantara University, Jakarta 11480, Indonesia; b Psychology Department, Faculty of Humanities, Bina Nusantara University, Jakarta 11480, Indonesia

**Keywords:** Indonesian telemedicine, Weighted sentiment analysis, Text classification, Telemedicine user review, Natural language processing, Indonesian language corpus, Health informatics

## Abstract

The digital era has fundamentally transformed the global healthcare paradigm. The World Health Organization (WHO) defines digital health as the utilization of information and communication technologies (ICT) to enhance the efficiency, accuracy, and accessibility of healthcare services. Consequently, Indonesia's e-health market is projected to reach US$ 3.6 billion by 2031. This growth is driven by rising digital literacy among the population, although infrastructural and regulatory challenges continue to demand serious attention. This phenomenon has generated a massive volume of user review data that contains highly valuable insights into user perceptions, experiences, and overall satisfaction with telemedicine services. Sentiment analysis of these reviews serves as a powerful tool for service providers to gather actionable feedback on their offerings. This paper introduces a novel dataset, named the Indonesian Telemedicine User Review Dataset for Sentiment Analysis. The dataset comprises over 19,680 rows of user reviews sourced from the three most prominent telemedicine applications in Indonesia. Two human annotators annotated the dataset, strictly adhering to guidelines rigorously validated by domain experts in Psychology. Specifically, the dataset contains 14,748 positive reviews, 711 neutral reviews, and 4,221 negative reviews. Ultimately, this dataset serves as a foundational resource to foster future innovations and advancements in sentiment analysis.

Specifications Table

**Table:** 

Subject	Computer Sciences
Specific subject area	*Natural Language Processing, Indonesian Language, User Review Classification*
Type of data	*Table* *Processed*
Data collection	User reviews were scraped from the Google Play Store using a Python library, covering Indonesian-language reviews of telemedicine applications (i.e., Alodokter, Halodoc, KlikDokter). Cleaning included the removal of personally identifiable information, missing values, and duplicates, as well as UTF-8 conversion. Reviews were manually annotated by two annotators with sentiment (Positive/Neutral/Negative) and weight (Uninformative/Informative/Complex) labels, following a guideline reviewed by a psychology expert. Inter-annotator agreement was measured with Cohen's Kappa; the expert adjudicated disagreements.
Data source location	*Telemedicine application user review (Alodokter, Halodoc, Klikdokter) on Google Play*
Data accessibility	Repository name: Mendeley Data Data identification number: 10.17632/rmmmf2dp8d.3 Direct URL to data: https://data.mendeley.com/datasets/rmmmf2dp8d/3 **License: CC BY 4.0** **Version: 3.0**

## 1 Value of the Data

• The Weighted Indonesian Telemedicine Sentiment Dataset contains 19,680 manually annotated Indonesian-language user reviews from three telemedicine applications, labeled with three sentiment classes (Positive, Neutral, Negative) and a weight attribute (Uninformative, Informative, Complex) indicating review informativeness.
• Two annotators followed documented guidelines created by a psychology expert, and inter-annotator agreement was measured using Cohen's Kappa (κ = 0.752), enabling other researchers to assess label reliability.
• This dataset introduces a weight attribute (Uninformative, Informative, Complex), which adds a crucial secondary dimension. This enables researchers to analyze not only what the user feels but also the substantive value of the review, thereby supporting more advanced tasks such as Aspect-Based Sentiment Analysis (ABSA). For example, analyze reviews with mixed emotions. On the other hand, this label can be used to curate data, such as filtering uninformative reviews or studying the characteristics of complex user feedback.
• This dataset facilitates and supports experiments in advanced NLP technologies, including BERT, LSTM, and LLMs, such as the user review sentiment classification task.

## 2 Background

The digital era has fundamentally transformed the global healthcare paradigm. The World Health Organization (WHO) defines digital health as the utilization of information and communication technologies (ICT) to enhance the efficiency, accuracy, and accessibility of healthcare services [1]. Consequently, Indonesia's e-health market is projected to reach US$ 3.6 billion by 2031. This growth is driven by rising digital literacy among the population, although infrastructural and regulatory challenges continue to demand serious attention [2,3].

Telemedicine platforms such as Halodoc, Alodokter, and KlikDokter have emerged as prominent digital healthcare providers, serving millions of users across Indonesia. As of 2024, Halodoc has 20 million users [4], generating a massive volume of user review data that contains highly valuable insights into users’ perceptions of telemedicine services. Users often struggle to select the platform that best aligns with their needs, particularly regarding pricing, service offerings, and the quality of interactions with medical professionals [5].

Sentiment analysis of these reviews serves as a powerful tool for service providers to gather actionable feedback on their offerings. However, applying sentiment analysis as a text classification task, categorizing text into positive, negative, or neutral labels within the context of the Indonesian language, is not straightforward. A clean dataset can facilitate experimentation for researchers in advanced sentiment analysis tasks.

## 3 Data Description

The dataset was collected from user reviews of telemedicine applications on the Google Play Store. It comprises over 19,680 reviews in Indonesian, encompassing both formal and informal registers, with some instances of code-mixed English. The entire dataset was manually annotated by two independent annotators.

The annotation process utilized three sentiment labels: Positive, Neutral, and Negative, based on criteria defined by Psychology domain experts, as detailed in Table 1. Each label has specific, measurable criteria. Positive sentiment encompasses reviews reflecting user satisfaction with the telemedicine application. Neutral sentiment includes reviews inquiring about health facts without conveying explicit or implicit positive or negative connotations. Conversely, negative sentiment comprises reviews expressing user disappointment or dissatisfaction with the telemedicine application's services.

**Table 1:** Sentiment annotation criteria.

Sentiment	Sentence Criteria	Sentence Example
POSITIVE	- Satisfaction with application - Appreciation for doctor about friendliness, accurate diagnosis, and appropriate drug prescriptions - Persuade to use application - Contains positive words like good, Brilliant, useful, or glad	*Penjelasan yang cepat dan tanggap dalam mengutamakan beberapa keluhan yang dialami* (quick and responsive in addressing and prioritizing the patient's reported complaints)
NEUTRAL	- Factual health question without implicit and explicit positive or negative sentiment	*Maaf dok saya punya riwayat gerd tapi sering nyeri di dada kiri kadang kanan tapi di kasih obat nitrokap aspilet dan yg lain itu sebetulnya gimana ya dok apakah ada masalah dengan jantung nya* (Sorry doc, i have a history of GERD but i often feel pain in my left chest, sometimes the right but i've been given medications like Nitrocap, Aspilet, and others. what's actually going on, doctor? is there a problem with my heart?)
NEGATIVE	- Disappointment with application - Complaint about doctor service - Contains bad word like bad, disappoint, or difficult	*Aplikasi gak guna, dokter chatnya gak dibalas,percuma uninstall aja* (Useless app, the doctor's chat not answered, pointless just uninstall it.)

**Fig. 1:**
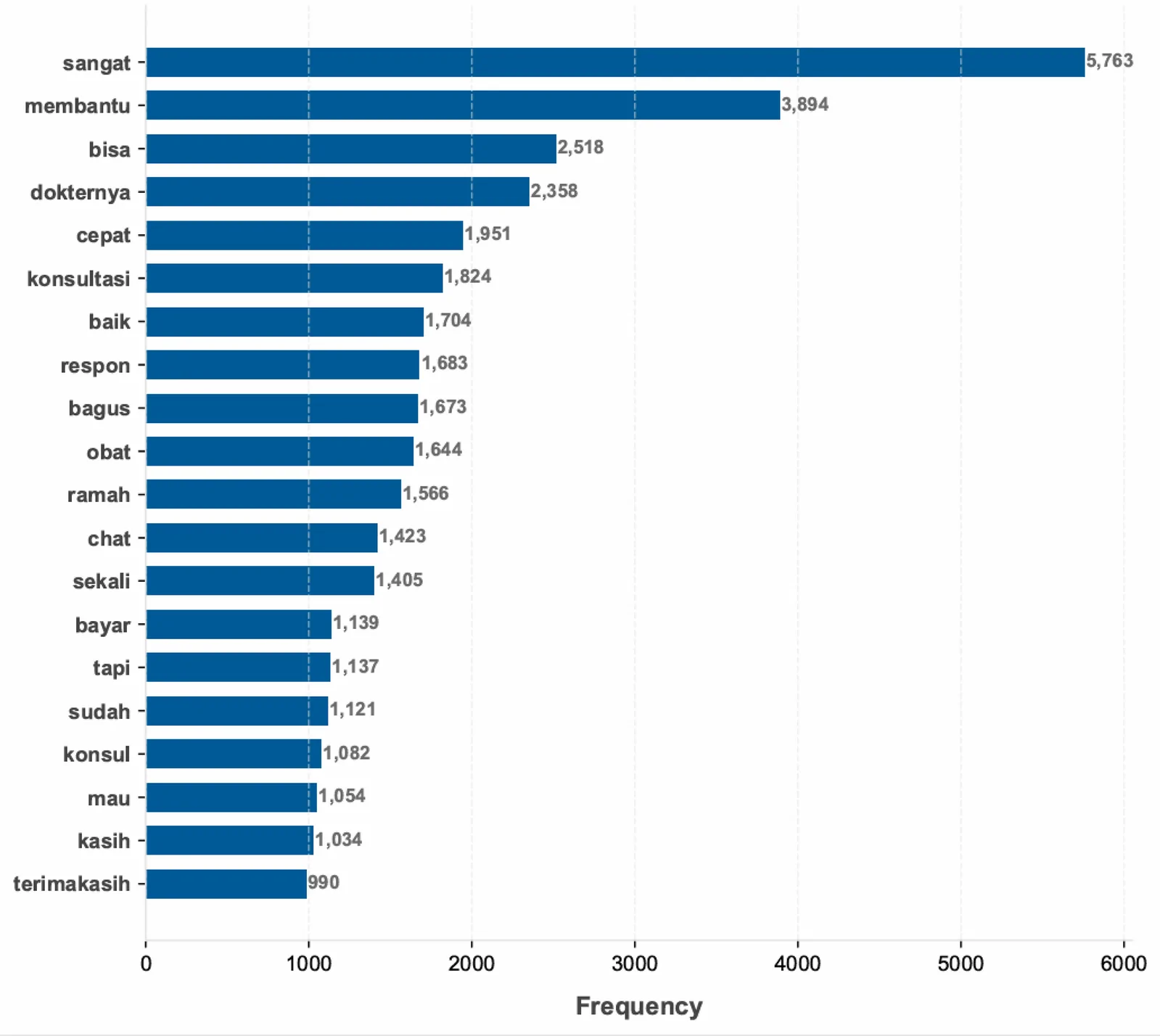
Top 20 most frequent words in the proposed telemedicine corpus.

This dataset is designed to support the development of automated classification models for user reviews in telemedicine applications. Fig. 1 illustrates an example of the dataset's use in a sentiment analysis task, while Table 2 describes the dataset attributes and their respective explanations.

**Table 2:** List of attributes and descriptions.

Attribute	Description
platform	Application distributor platform
app_name	Application name
rating	Application user satisfaction score
review	Application user review
sentiment	Data label (negative, neutral, positive)
weight	User review complexity label (uninformative, informative, complex)

The review and sentiment attributes serve as the primary features for training and evaluating the models. Additionally, the authors include the rating attribute as supplementary data, which can be combined with the review attribute in future studies to enhance the model's sentiment classification capability. The authors also introduced a weight attribute that researchers can use to curate or filter reviews based on the criteria outlined in Table 3. Importantly, to maintain user privacy, this dataset strictly excludes any personally identifiable information (PII), such as usernames.

**Table 3:** Weight annotation criteria.

Weight	Sentence Criteria	Sentence Example
UNINFORMATIVE	- Contains 1 word or short sentence - Emotion expression without explainability - Generic Sentence	*Kecewa* (Dissapointed)
INFORMATIVE	- Complete Sentence - Formal or Semi-Formal Language - Easy to classify the sentiment	*Pelayanan dokter sangat baik* (The doctor’s service is very good)
COMPLEX	- Mix Indonesia language and other languages - Contains slang, non-formal language, or abbreviation - Contains mixed emotions	*Aplikasinya bagus, tetapi dokternya ga emphatic* (The application is good, but the doctor not empathic)

Beyond sentiment distribution, the dataset exhibits distinct statistical characteristics. To provide a comprehensive overview of the dataset's composition and textual properties, we present the detailed descriptive statistics in Table 4.

**Table 4:** Descriptive statistics of the dataset.

Metric	Value / Description
Total Reviews	19,680
Sentiment Distribution	Positive: 14,748 (74.9%) Neutral: 711 (3.6%) Negative: 4,221 (21.5%)
Weight Distribution	Uninformative: 16,572 (84.2%) Informative: 2,718 (13.8%) Complex: 390 (2.0%)
Average Review Length	11.8 words
Median Review Length	7 words
Standard Deviation (Length)	12.9 words
Min / Max Length	1 word / 111 words
Total Vocabulary Size	23,157 unique tokens

**Fig. 2:**
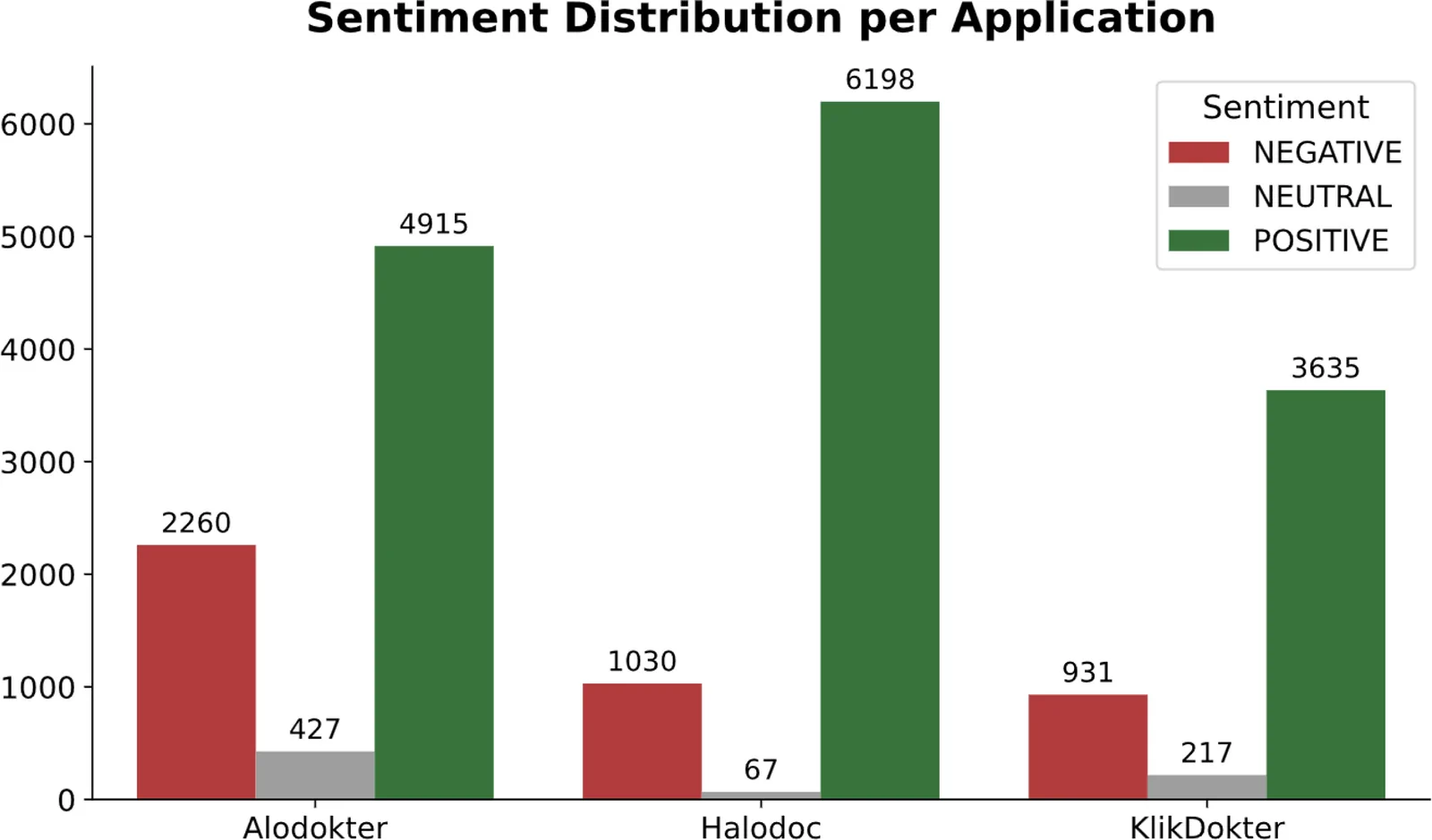
Sentiment distribution of user reviews across telemedicine applications.

**Fig. 3:**
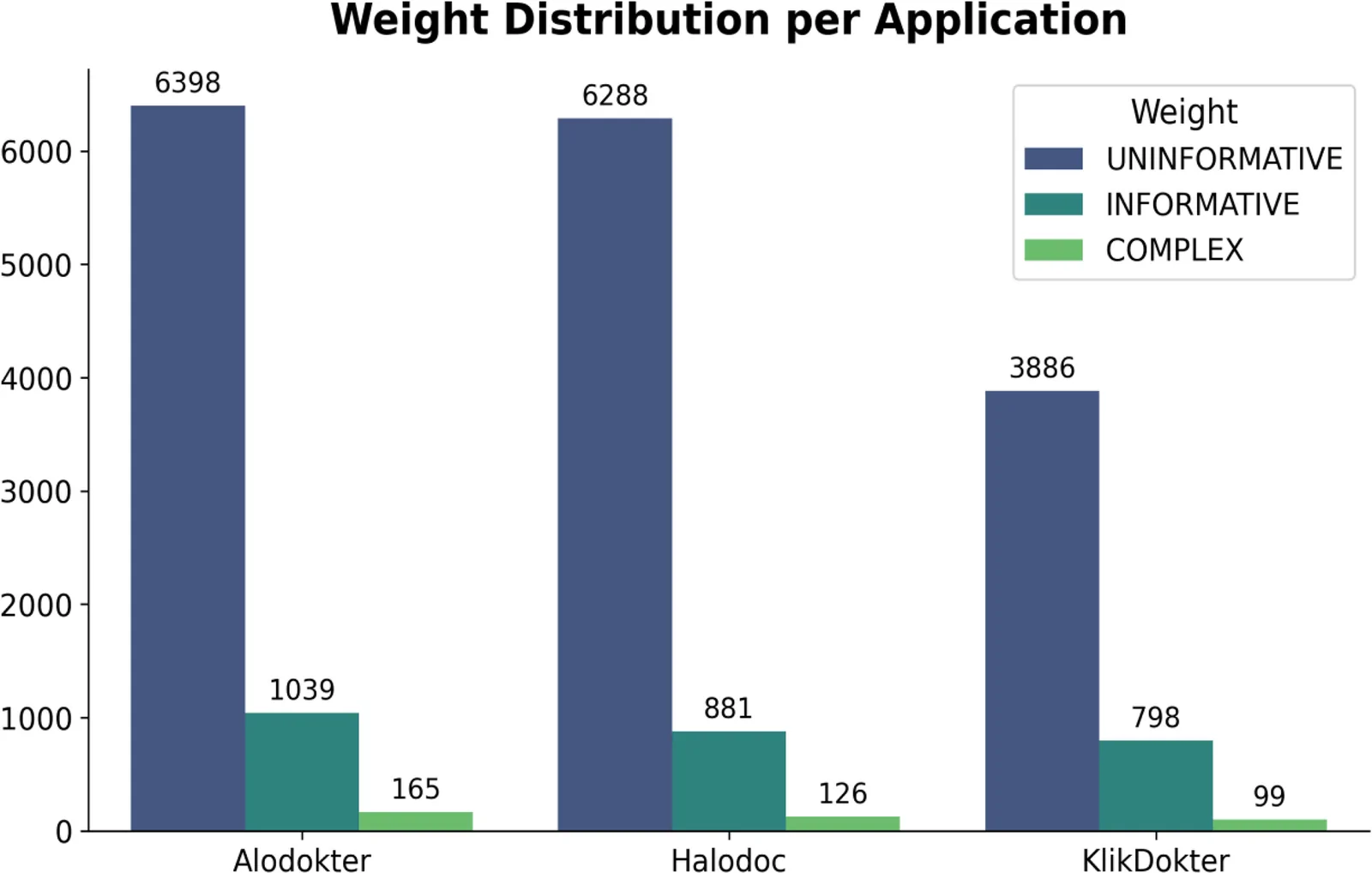
Distribution of informativeness weight categories (uninformative, informative, complex) across telemedicine applications.

The dataset is publicly accessible via Mendeley Data and stored in CSV (comma-separated values) format. The data collection and annotation process yielded a final dataset of 19,680 labeled reviews, with the following label distribution: 14,748 positive, 711 neutral, and 4,221 negative. The sentiment distribution for each telemedicine application is shown in Fig. 2, and the distribution of the weight attribute across applications is shown in Fig. 3.

## 4 Experimental Design, Materials, and Methods

This study presents an updated and refined version of a previously published telemedicine review dataset [6]. While the previous iteration focused on a larger volume of binary (positive/negative) classifications, the current version introduces significant methodological and structural enhancements. To better demonstrate the novelty and scientific contribution of this work, Table 5 highlights the key differences between our current dataset and other existing Indonesian sentiment datasets. Unlike general mobile app datasets [7] or automated government app reviews [8], this dataset is strictly curated for the telemedicine domain. Furthermore, it is the first to incorporate a validated weight attribute alongside a three-class sentiment system, with annotations rigorously validated by domain experts in Psychology to ensure clinical and contextual relevance [9].

**Table 5:** Comparison of related datasets and the proposed telemedicine corpus.

Dataset & Reference	Sentiment Classes	Total Reviews	Domain	Weight / Informativeness Attribute	Annotation & Validation Method
Indonesian Healthcare App Reviews [10]	2 (Positive, Negative)	9,310	General Healthcare	No	Basic manual / Heuristic
Sentiment analysis of user review dataset in Indonesia [6]	2 (Positive, Negative)	255,679	Telemedicine	No	Manual (Majority voting)
Multilabel Sentiment & Emotion Dataset [7]	3 (Positive, Neutral, Negative) + 6 Emotions	21,697	General Mobile Apps	No	Manual annotation
IGAR: Government App Review Dataset [8]	3 (Positive, Neutral, Negative)	617,722	Government (incl. Health)	No	Automated (Rating-based & VADER)
A Weighted Sentiment Dataset for Indonesian Telemedicine Text Classification (This Work)	3 (Positive, Neutral, Negative)	19,680	Telemedicine	Yes (Uninformative, Informative, Complex)	Manual + Psychology Expert Validation + Cohen’s Kappa (0.752)

**Fig. 4:**
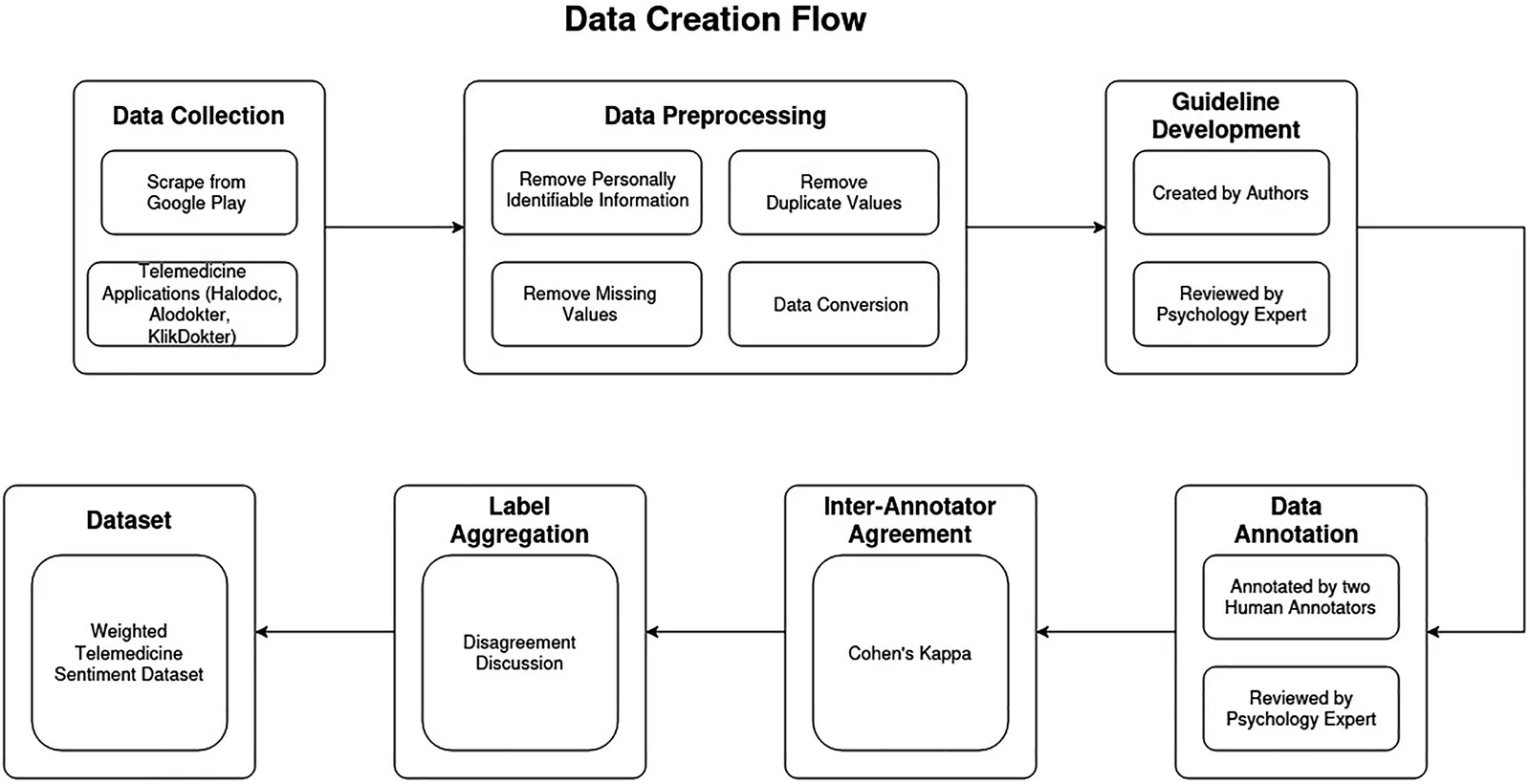
Data creation flow.

The dataset development process was carried out in seven systematic stages (see Fig. 4): Data Collection, Data Preprocessing, Guideline Development, Data Annotation, Inter-Annotator Agreement, Label Aggregation, and Final Dataset.

The dataset was collected from the Google Play Store distribution platform using web scraping with Python libraries between May 15 and June 15, 2026 [15]. Mining app reviews has proven effective for capturing fine-grained user needs and sentiments [11]. The collected data comprises user reviews in Indonesian, with code-mixing with other languages, such as English. Data collection focused specifically on reviews of three telemedicine applications operating in Indonesia: Alodokter, Halodoc, and Klikdokter.

Once the dataset was obtained, a series of data-cleaning processes were conducted to ensure data quality, integrity, and ethical use [12]. The cleaning stages included: (1) the removal of attributes containing Personally Identifiable Information (PII) to guarantee anonymity; (2) the elimination of rows with missing values; (3) the removal of duplicate data; and (4) the conversion of data encoding to UTF-8 format to prevent anomalous symbols from disrupting the analysis process.

Prior to the annotation process, an annotation guideline was developed that encompassed detailed criteria for each sentiment label and the weight attribute (see Table 1, Table 3). This guideline aimed to ensure uniformity of perception and criteria among all annotators, a crucial step in minimizing subjective bias [13]. The developed guideline was subsequently reviewed by Psychology experts to validate that the established criteria aligned with applicable scientific principles.

The dataset was manually annotated by two annotators. A fundamental difference from the previous dataset lies in the manual annotation process, which was validated by Psychology experts, ensuring that the resulting annotations align with scientific psychological principles. In cases where annotators assigned different labels to the same review, a discussion was held to reach consensus. If consensus could not be reached, the disagreement was resolved through consultation with a Psychology expert. In addition to being assigned sentiment labels (Negative, Neutral, and Positive) according to the annotation guideline, each review in this dataset was also weighted based on its complexity level. The weight labels consisted of three categories: Uninformative, Informative, and Complex, which were also validated by Psychology experts.

The dataset, now labeled with sentiment and weight, subsequently underwent a voting process via a peer-review mechanism to evaluate the quality of the annotations. Inter-annotator agreement was calculated using Cohen's Kappa, a reliable standard metric for measuring the reliability of categorical data annotation [14], yielding a score of 0.7520. Furthermore, to ensure the accuracy of the annotated data, the authors were assisted by Psychology experts to manually validate a sample of the annotation results.

## Limitations

The published dataset presents a few limitations that warrant consideration. First, user reviews for the three selected applications were sourced exclusively from a single application distribution platform: the Google Play Store. While this platform was selected for its dominant market share and largest user base in Indonesia, reviews from other distribution platforms (such as the Apple App Store) were not captured. Consequently, the dataset does not encompass all user feedback across all platforms. Furthermore, the distribution of sentiment labels within the dataset is inherently imbalanced. This class disparity may lead to model oversampling or bias toward positive sentiment during training. To mitigate this limitation, we introduced a weight attribute. This feature enables future researchers to curate and filter user reviews using assigned weights, thereby facilitating the construction of a more balanced sentiment distribution tailored to specific modeling requirements.

Second, the dataset is restricted to user reviews from only three telemedicine applications: Alodokter, Halodoc, and KlikDokter. Although the Indonesian market features a wide array of telemedicine applications, these three were specifically chosen because they possess the largest user bases. Consequently, this selection ensures a highly diverse and comprehensive corpus of user reviews, providing a robust and representative foundation for analysis despite excluding smaller or emerging platforms.

## Ethics Statement

This study involved data collected from a public online platform (Google Play Store). In compliance with ethical guidelines, the authors confirm that:

a) Explicit informed consent was not obtained as the data consists of publicly available user reviews. During the data cleaning process, all attributes containing personally identifiable information (PII) were strictly removed to ensure user privacy and confidentiality.
b) The data collection and redistribution processes complied with the Google Play Store’s Terms of Service^1^ and data redistribution policies^2^. The resulting dataset contains no sensitive personal information and is intended solely for academic and research purposes.

## Credits Author Statement

**Fakhri Wahid Athallah**: Conceptualization, Methodology, Visualization, Writing – Original Draft. **Ihya Choerul Ulumudin:** Data Curation, Methodology, Conceptualization. **Rhio Sutoyo**: Conceptualization, Methodology, Validation, Writing – Review & Editing, Supervision. **Esther Widhi Andangsari**: Methodology, Conceptualization, Validation.

## Declaration of Competing Interest

The authors declare that they have no known competing financial interests or personal relationships that could have appeared to influence the work reported in this paper.

## Footnotes

1 https://play.google.com/about/play-terms/.

2 https://support.google.com/googleplay/android-developer/answer/10144311.
